# Fruit Juices: Are They Helpful or Harmful? An Evidence Review

**DOI:** 10.3390/nu13061815

**Published:** 2021-05-27

**Authors:** Carrie H. S. Ruxton, Madeleine Myers

**Affiliations:** 1 Nutrition Communications, East Road, Cupar KY15 4HQ, UK; 2 Non-Diet Nutrition, Shirenewton NP16 6RL, UK; madi@nondietnutrition.co.uk

**Keywords:** fruit juice, cardiovascular, type 2 diabetes, obesity, nutrient density, cognitive function, polyphenols, potassium

## Abstract

Dietary guidelines often deal with 100% fruit juice (FJ) inconsistently because it represents a source of free sugars. However, FJ also provides bioavailable micronutrients and plant bioactives at levels similar to those found in whole fruits. The present review weighs up the evidence from high-quality studies investigating a potential health harm for FJ against evidence from studies which indicate a potential health benefit. The findings reveal that FJ consumption, at moderate intakes consistent with the dietary guidelines for the US and some European countries (75–224 mL daily), does not increase the risk of obesity, type 2 diabetes, cardiovascular disease or poor glycaemic control. In contrast, regular consumption of FJ—even up to 500 mL per day in short-to-medium-term studies—appears to confer a health benefit in terms of vascular function and reduced blood pressure. Emerging evidence for cognitive health benefits requires further investigation in human trials. Observational studies report associations between FJ and nutrient adequacy and suggest that FJ consumption is associated with reduced risk of stroke. In conclusion, FJ appears to offer more benefit than risk and there appears to be no justification for discouraging FJ within a balanced diet for children and adults.

## 1. Introduction

Fruit juice (FJ) is a drink made from the extraction or pressing of the natural liquid contained in fruit and vegetables. While it is a source of free sugars, it also contains micronutrients and plant bioactives. In countries, such as the UK, Spain, Austria, Denmark, France, Hungary and Ireland, one serving of FJ counts towards daily recommendations for fruits and vegetables [1]. The Dietary Guidelines for Americans, 2020–2025 allow for up to half of the recommended daily fruit intake to be replaced by FJ [2]. In contrast, in other countries such as the Netherlands, FJ is classed as a sugary drink and discouraged [3]. A review of country-based food-based dietary guidelines found that, while 7% of guidelines were negative about FJ, only 23% clarified their role in a balanced diet while 38% failed to provide any specific guidance on FJ [4]. Therefore, it is difficult for consumers to navigate these conflicting messages to determine, firstly, whether FJs are helpful or harmful in a health context and, secondly, how much can be consumed in a balanced diet. This is compounded by the differing serving sizes noted in public health guidance (80–237 mL) versus the broad range of intakes (200–750 mL) used in scientific studies.

FJ is strictly defined in law and must meet specific requirements relating to its production and composition. The 2001 EU Fruit Juice Directive defines FJ as: “The fermentable but unfermented product obtained from the edible part of fruit which is sound and ripe, fresh or preserved by chilling or freezing of one or more kinds mixed together having the characteristic colour, flavour and taste typical of the juice of the fruit from which it comes” [5]. In the EU and UK, it is not permitted to add sugar to FJ and the Brix level (concentration): “shall be the one of the juice as extracted from the fruit and shall not be modified, except by blending with the juice of the same species of fruit” [5]. This means that the sugar content of FJ cannot be further modified and is representative of the sugar content of the intact fruit.

Assumptions made about the risks of consuming FJ include a potentially negative impact on cardiovascular health, body weight/body mass index, glycaemic control and risk of type 2 diabetes. There are also beliefs that FJ may be favoured over whole fruit, or consumed in excessive amounts. Concerns focus on the naturally occurring sugars in FJ which are classified as free sugars by the World Health Organisation [6]. The classification was based on an assumption that 100% of the sugars in FJ exist outside the intact cell wall although this does not appear to have been confirmed in practice [7].

The purpose of this review is to weigh up the evidence from studies investigating a potential health harm for FJ (in relation to obesity, metabolic health and cardiovascular disease [CVD]) against evidence from studies which indicate a potential health benefit (in relation to cardiovascular health, nutrient adequacy and cognitive function). It will also be considered whether the current evidence is sufficient to indicate optimal intakes of FJ, which would help inform public health messages.

## 2. Material and Methods

As several systematic reviews and meta-analyses (SRMAs) examining FJ and specific health outcomes have been published in the past 3 years and the focus of this review was to weigh up risks and benefits from a public health perspective, conducting another SRMA was outside the scope of this paper. Instead, the literature search strategy in PubMed (covering January 2001–April 2021) focused on including all SRMAs with the keyword ‘juice’ and then excluding those which were unrelated to fruit juices, e.g., ‘gastric juice’, ‘beetroot juice’, or which reported health outcomes not relevant to the target topics: obesity/weight gain, CVD, glycaemic control/risk of type 2 diabetes.

Where no SRMAs were available, the search strategy switched to all randomised controlled trials (RCT) which related to the target health topics with the keywords ‘fruit juice’ OR ‘orange juice’ OR ‘grapefruit juice’ OR ‘pomegranate juice’ OR ‘apple juice’. References of papers were also searched to identify additional RCTs. Prospective observational studies were only included where no SRMAs or RCTs were published on the reviewed topics—this applied to nutrient adequacy and obesity/weight gain in children.

A limitation of this approach is that there is a risk that individual studies may have been missed. However, the risk is low given the recent publication of several SRMAs, which would have conducted a thorough, systematic search of the literature.

## 3. Nutritional Composition, Bioavailability and Intakes

### 3.1. Composition

The free sugars classification does not differentiate between naturally occurring sugars in FJ and those added to foods and beverages, hence there is an implication that all food sources pose a similar risk of harm, which may not be the case given their differing nutritional values.

As Table 1 shows, FJ have a nutritional composition that is more similar to whole fruit than to sugar-sweetened beverages (SSBs). One major difference between the juice and fruit is the higher pectin (fibre) content of whole fruit, which is largely removed during manufacture to produce juice. Nevertheless, when expressed as a typical serving, the difference in fibre between the edible part of a whole orange (80 g) and a small glass of orange juice (150 mL) is approximately 1 g [8], equating to just 3% of the EU/UK Dietary Reference Value of 30 g.

The phytonutrient and micronutrient content of FJ, which sets it apart from SSBs that contain negligible nutrition beyond carbohydrates and energy, could theoretically confer health benefits. The range of plant bioactive compounds provided by FJ is broad. For example, apple juice contains chlorogenic acid, quercetin and catechins; orange juice is rich in hesperidin, narirutin, carotenoids and hydroxycinnamic acids, while blood orange juice is also a source of anthocyanins [13,14,15]. Dark coloured berry and grape juices contain anthocyanins, as well as various phenolic acids and stilbenes and pomegranate juice contains punicalagin and punicalin [16,17]. FJ is recognised as making a significant contribution to intakes of polyphenols; one study estimated that citrus fruit and juices combined were the greatest contributors to flavanone intake in all regions of Europe studied [18].

### 3.2. Bioavailability

Nutrients in a food mean little unless they are available to the body. Consistent evidence from blood biomarker studies confirms that the vitamins, minerals and bioactives in FJ are available to be absorbed by the body [9,15,19].

In a 3-week study, 13 healthy, normal-weight adults drank 236 mL orange juice three times daily [20]. Compared with baseline levels, vitamin C and folate levels increased by 59% (*p* value < 0.001) and 46% (*p* value = 0.018), respectively, while polyphenol levels rose by eight times (*p* value = 0.045) and carotenoids increased by 22% (*p* value < 0.001).

Two RCTs which compared fresh and juiced fruit showed that β-cryptoxanthin, hesperidin and narirutin were significantly more bioavailable and bioaccessible from 100% orange juice than they were from whole oranges—even though the whole fruit has higher levels of these potentially beneficial plant compounds [9,15]. This could be due to the higher pectin levels in the whole fruit inhibiting absorption, or because juicing breaks down cell walls within the fruit, leading to a greater release of polyphenols. Other work has found that more of the carotenoids in orange juice are present in droplet form which may explain why absorption from juice is greater than from whole fruits [21].

When comparing packaged versus freshly squeezed orange juice, approximately three times more hesperetin (a metabolite of hesperidin) appears in blood after consumption of packaged orange juice [22]. The authors explained this difference by confirming that the more efficient commercial juicing process creates a richer hesperidin content.

### 3.3. Intakes in Europe

According to market data, the average EU citizen drank 11.5 litres of FJ during 2018, equating to 32 mL per day [23]. This is a modest intake compared with other beverages, e.g., sugar-sweetened beverages, as well as recommended serving sizes (80–200 mL). Therefore, contrary to concerns, FJ does not appear to be overconsumed on average.

There is considerable variation of intakes across European countries, for example 37% of UK adults report some FJ consumption compared with 84% of Dutch and 49% of French [24,25,26]. Intakes in adult consumers also vary, with 40 mL consumed daily in Netherlands compared with 55 mL in France and 130 mL in the UK [24,25,26].

## 4. Potential Risks of Fruit Juice Consumption

### 4.1. Type 2 Diabetes

Observational trials report that SSBs are associated with increased risk of type 2 diabetes and poor glycaemic control [27,28,29,30,31]. This has led to speculation that FJ represent a similar risk for type 2 diabetes on account on their sugar content. However, the evidence does not support this view. The UK’s Scientific Advisory Committee on Nutrition examined the evidence for carbohydrates and health, which included commentary on FJ [32]. While consumption of SSBs was statistically associated with risk of type 2 diabetes, similar links were not seen for FJ. Supporting this view, a SRMA of three cohorts with 137,663 participants concluded that FJ consumption was not statistically associated with the risk of developing type 2 diabetes (RR = 1.03, *p* value = 0.62) [33]. This has been confirmed by an updated SRMA based on five cohorts with 320,014 participants [34].

A recent SRMA looking at the dose–response relationship between FJ and metabolic syndrome—associated with increased risk of type 2 diabetes—found a U-shaped association, with protection at moderate intakes of FJ (RR for 125 mL/d = 0.77) [35]. A U-shaped curve suggests negative effects at both very low and excess intakes, whereas linear associations, as reported for SSBs, suggest increased negative effects with increasing intakes [35]. Studies have attempted to demonstrate the importance of separating SSBs and 100% FJ when looking at dose–response effects, because of the many differences between the beverages [36].

### 4.2. Glycaemic Control

A SRMA combining 18 RCTs in 960 participants reported that FJ consumption had no significant impact on fasting blood glucose and insulin, insulin resistance, or glycated haemoglobin [37]. Many of the participants in this analysis were overweight or had existing metabolic risk factors, such as raised cholesterol or type 2 diabetes. A second SRMA combined 12 RCTs in 412 participants, including those who were obese or had underlying risk factors for type 2 diabetes or CVD [38]. In half of the studies, daily FJ intakes were in excess of 400 mL but, despite this, FJ consumption was not found to significantly impact fasting blood glucose or insulin levels. A third SRMA of 155 study comparisons (5086 participants) concluded that certain food groups, including SSBs and sweetened milks, had reported ‘harmful effects’ on glycaemic control when tested in in substitution studies [39]. In contrast, both fruits and FJ were reported as offering beneficial effects. A fourth SRMA found no impact of regular FJ consumption on blood glucose or insulin levels, HOMA index, or HbA1c based on data from 3 to 23 RCTs [34].

Why have SRMAs reported null effects on glycaemic control considering that FJs are a source of free sugars which are, from a WHO standpoint, no different from those added to SSBs? Two reasons have been proposed. Firstly, FJs are classified as low glycaemic index (GI), suggesting that they have a smaller impact on post-prandial blood glucose levels than a reference food. For example, 100% apple juice has a GI of 41 (whole apples = 36) while 100% orange juice has a GI of 50 (whole oranges = 43) [40]. This compares favourably with an average SSB which has a GI of 63–68.

A second reason is linked to the polyphenol content of FJ which has been proposed to have a “double action” on glycaemia. Studies have found that polyphenols, such as hesperidin in citrus juice or punicalagin and punicalin in pomegranate juice, slow down post-prandial glucose absorption from the gut resulting in a flatter post-prandial blood glucose peak [17,41]. The 2019 study by Kerimi et al., found that hesperidin influences the actions of GLUT2 and GLUT5 sugar transporters, leading to a significantly lower rate of glucose uptake. This could potentially involve the polyphenol metabolites produced by the gut microbiota, which have an effect much later than the initial post-prandial period [17].

### 4.3. Obesity and Excess Weight Gain

A typical 150 mL serving of FJ contains approximately 60 kcal, equivalent to 3% of the EU Reference Intake for energy. Nevertheless, FJ consumption has been blamed by some commentators for contributing to obesity [42]. Inconsistent findings have been reported by observational studies, perhaps because these rely heavily on self-reports of beverage consumption which are vulnerable to error and bias, and often combine FJ with sugar-sweetened fruit drinks [43,44]. Therefore, a more reliable assessment of the impact of FJ on weight gain and obesity risk is to include evidence from intervention trials.

#### 4.3.1. Studies in Adults

Two SRMAs have examined the impact of FJ on obesity risk and body composition in adults. D’Elia et al., 2020 found no statistical associations between regular FJ consumption and body mass index or weight gain using data from observational studies and RCTs [34]. A similar conclusion was reported by a second SRMA which included measures of body weight, body mass index, waist circumference, fat mass or lean body mass from RCTs [45]. It is noteworthy that, out of 17 human intervention trials where 100% orange juice was consumed at intakes of 250–750 mL daily for 4 to 12 weeks, none reported a statistically significant change to body weight [46].

This does not mean that large intakes of FJ can be encouraged since two interventions found adverse effects on short-term anthropometric markers at the extremes of consumption. A 2-week RCT found that consuming 20% of daily energy requirements as FJ between meals resulted in a statistically significant 1 kg increase in fat mass, while the same amount of FJ consumed with meals significantly reduced fat mass by 0.3 kg [47]. A second trial required healthy lean and overweight adults to consume either whole fruit, vegetables or FJ, equating to 20% of daily energy requirements [48]. On average, participants gained weight and fat mass over the 8-week intervention, with no statistically significant difference between the fruit/vegetable and FJ groups, but considerable individual variation in body fat change in overweight and obese participants. In assessing these trials, it is evident that the 980–1300 mL daily consumption of FJ was not fully compensated by a reduction in other foods, resulting in an overall energy gain which led to accretion of fat mass. In contrast, a more modest daily intake of 150–300 mL FJ, as part of a 12-week intervention to boost fruit and vegetable consumption to 8 servings per day, did not lead to a statistically significant change in body weight [49].

#### 4.3.2. Studies in Children

There is a distinct lack of RCTs in children looking at the impact of FJ on weight gain, highlighting an important evidence gap. Just one 12-week RCT was found which was designed to test the nutritional impact of three fortified orange juices in 180 children with a mean age of 8 years [50]. The authors noted that “body weight remained as expected for growth” during the study, indicating that the twice-daily glass of 240 mL did not have an adverse effect on weight change.

One SRMA was located by Auerbach et al. [51] which included 8 prospective cohort studies involving more than 34,000 children. This found no association when all studies were combined but different associations between FJ consumption and body mass index z-scores for younger versus older age groups. The authors concluded: “Consumption of 100% fruit juice is associated with a small amount of weight gain in children ages 1 to 6 years that is not clinically significant, and is not associated with weight gain in children ages 7 to 18 years”. Daily intake ranges of FJ in the different age groups differed markedly with younger children consuming 224–504 mL and older children consuming 109–190 mL. Controlling for energy intake did not affect the outcomes of the Auerbach study [51] but resulted in the association between consumption of FJ and weight/body fatness becoming non-significant in a systematic review of 22 studies in children and adolescents [52].

In a prospective study of more than 500 British children, no association was found between FJ consumption and body fatness at age 5 or 7 years [53]. A second prospective study followed up 100 US children from the age of 3–6 years, finding that regular FJ consumption was not associated with changes in body mass index but did predict higher whole fruit consumption in adolescence [54]. A third prospective study followed 7301 children aged 9–16 years for two years on average [55]. Orange juice consumption was not associated with body mass index percentile change; with the highest consumption group drinking more than 1 glass daily. Therefore, apart from the non-clinically significant weight gain seen in 1 to 6 year olds [51], drinking FJ does not appear to promote excess weight/fat gain in children.

### 4.4. Evidence for Safe Intakes

The current evidence in adults suggests that drinking FJ on a regular basis, even daily, is not a cause of weight gain nor does it influence the risk of obesity. RCTs which investigated daily FJ intakes of 250–750 mL for 4 to 12 weeks recorded no significant weight gain, despite these levels being in excess of current guidance. There is now a need to examine the impact of regular FJ consumption over a longer time period, e.g., 1 year.

In children, the only available SRMA was based on observational studies and indicated that 224–504 mL of FJ daily in younger children was associated with a small but statistically significant risk of excess weight gain [51]. In contrast, the more modest intakes in older children (109–190 mL daily) were not linked with excess weight gain. The only RCT located tested 480 mL daily for 12 weeks, finding no adverse effects on body weight [50]. The longitudinal study by Wan et al., 2020 found that consumption of 1.5 cups daily (equivalent to 224 mL) was not associated with excess weight gain [54]. The limited evidence from these studies suggests that intakes below 224 mL (8 oz) daily would be appropriate for school-aged children. A US expert committee concluded that FJ should be limited to children aged over 12 months at age-appropriate intakes ranging from 4–8 oz (112 mL to 224 mL) per day [56].

Turning to type 2 diabetes and glycaemic control, two SRMAs suggested no risk of type 2 diabetes at FJ intakes of 75 and 150 mL or up to 220 mL [34,35]. The SRMA that included RCTs [34] found no negative impact on glycaemic control at daily FJ intakes of 323–430 mL.

## 5. Potential Benefits of Fruit Juice Consumption

### 5.1. Associations with Nutrient Adequacy

No SRMAs were found which examined associations between FJ consumption and nutrient/diet adequacy. Individual observational studies tend to show that FJ consumption is typically associated with higher nutrient density or better-quality diets, when compared with diets where FJ is not consumed, or only at low levels [24,26,52,57,58,59,60]. However, bearing in mind the limitations of observational studies, particularly that FJ consumption may simply be a marker of healthier habits, it cannot be assumed that drinking FJ improves nutrient adequacy, only that it is associated with higher-quality diets which may indicate a general healthier lifestyle in FJ consumers.

A French study found that FJ consumers tended to eat more whole fruits and vegetables than non-consumers and had significantly greater intake of fibre (18.6 g (SEM 0.2) vs. 19.8 g (SEM 0.3) (*p* value < 0.0001)) [59]. This and other studies [54] suggest that FJ is not replacing whole fruit or vegetables in the diet or being a marker of low fibre intakes; common concerns about FJ consumption.

Additional evidence comes from two clinical trials where daily FJ formed part of the intervention. The first, in 45 healthy adults with habitually low fruit/vegetable intake, examined the nutritional and metabolic impact of consuming 8 daily servings of fruit and vegetables, including 150–300 mL of FJ [49]. After 12 weeks, significant rises were seen in blood levels of vitamin C (35%), folate (15%) and carotenoids (50–70%) compared with participants following their habitual diets. Average daily total sugars increased by 37 g in the intervention group, 20 g of which were free sugars and likely to have come from the FJ. Despite the significant sugar increase, overall energy intakes remained constant across the intervention, as did body weight. A second trial evaluated the impact of an energy-reduced diet in 78 obese women, with half consuming 500 mL of orange juice daily between meals while the remaining participants ate an energy-matched snack [61]. Following the 12-week intervention, blood levels of vitamin C rose by 62% (*p* value ≤ 0.015), while blood folate rose by 39% (*p* value = 0.033).

Although polyphenols are not classed as nutrients there is a wealth of evidence supporting their intake for lowering risk of disease [62]. FJ provides a range of potentially beneficial polyphenols, including anthocyanins, flavonols, flavan-3-ols and flavanones, and several types of juice are rich in polyphenols, contributing significantly to the polyphenol content of the diet [63,64]. The observational study by Zamora-Ros et al., 2011 estimated flavonoid intake and dietary sources using data from 36,037 participants and found that citrus-based juices were one of the major food sources of dietary flavanones [18]. Apple products (including apple juice) are estimated to be among the top 3 or 4 dietary sources of total polyphenols consumed in the USA [65,66].

### 5.2. Cardiovascular Health

Several trials have found that FJ intake contributes to blood pressure (BP) reduction. A SRMA of 19 RCTs comprising 618 participants reported that FJ consumption reduced diastolic BP by 2.07 mmHg (*p* value = 0.02) on average compared with a placebo [67]. The authors estimated that such a reduction could lower the incidence of hypertension by 17% and coronary heart disease (CHD) by 6%. However, no other significant effects on other markers of CVD risk were found. This is supported by a SRMA of more than 80 cohort studies which followed up 4,031,896 individuals for an average of 11 years [68]. During that time, more than 125,000 cardiovascular events were recorded. The study found that fruit, vegetables and FJ were similarly associated with a relative risk reduction of 13–14% in heart disease mortality. Additionally, regular FJ consumption was statistically linked with a 33% reduced risk of stroke mortality; greater than the risk reduction seen for whole fruit (13%) and vegetables (6%). The study concluded that the most important individual fruits and vegetables for lowering the risk of CVD were citrus fruits, FJ, apples, allium vegetables, carrots, cruciferous vegetables and green leafy vegetables.

A third SRMA evaluated the evidence from 21 observational studies and 35 RCTs [34]. Regular consumption of FJ was associated with a statistically significant lower risk of stroke (up to 22% depending upon intake) and a significant reduction in cardiovascular events. Looking specifically at RCTs, drinking FJ significantly lowered BP (systolic BP: −3.14 mmHg; diastolic BP: −1.68 mmHg) and significantly improved vascular function (carotid-femoral pulse wave velocity: −0.38 m/s; flow-mediated dilation (FMD): 2.1%), which would be expected to impact favourably on stroke risk. There was no impact of FJ consumption on blood lipids or inflammation.

A fourth SRMA of 8 RCTs reported that pomegranate juice reduced systolic BP on average by 4.96 mmHg (*p* value < 0.001) and diastolic BP by 2.01 mmHg (*p* value = 0.021) compared with placebos [69]. A fifth SRMA of 10 RCTs evaluating orange juice consumption and CVD and metabolic risk factors found a significantly beneficial effect of drinking orange juice (approximately 500 mL daily) on glucose, insulin, total cholesterol, low-density lipoprotein cholesterol and inflammation [45]. A sixth SRMA of 16 RCTs comprising 572 subjects reported that pomegranate juice significantly reduced some markers of inflammation compared with placebo (hs-CRP: −6.57 mg/L, *p* value < 0.001; IL-6: −1.68 pg/mL, *p* value < 0.001; TNF-α: −2.37 pg/mL, *p* value < 0.01) [70]. Chronic inflammation is a marker of enhanced CVD risk.

Two recent RCTs were not included in these SRMAs. In a crossover trial, 15 healthy overweight adults drank 400 mL daily of blood orange juice or a sugar-matched control drink with meals [71]. After 2 weeks, FMD improved by 2% (*p* value = 0.002), reported to be a clinically significant finding in relation to CVD risk reduction. Data on urinary excretion of citrus flavanone metabolites provided evidence that the FMD increase was related to hesperidin—the major polyphenol found in orange juice. A second RCT in 159 adults with raised systolic BP compared regular orange juice with a hesperidin-enriched orange juice and a sugar-matched placebo; all 500 mL daily [72]. After 12 weeks, there were significant improvements in BP and pulse pressure in both the regular and enriched orange juice, with a dose–response effect seen for hesperidin content.

#### 5.2.1. Mechanisms of Action

It has been proposed that the positive vascular effects seen for FJ consumption could be due to potassium, which has a claim in the EU for supporting normal BP, as well as vitamin C, nitrates, folate and polyphenols [73]. Higher dietary potassium intake is associated with lower rates of stroke and may also reduce the risk of CVD and CHD [74]. Several types of FJ provide an amount of potassium that would be classified as a significant amount according to European Law (≥7.5% per 100 mL). Per serving of 150 mL, orange juice provides 228 mg potassium; grapefruit juice 150 mg; tomato juice 345 mg and pomegranate juice 348 mg [11,75].

The polyphenols in FJ have also been proposed to be involved in specific beneficial effects on inflammation and vascular function. Citrus flavanones have been implicated in inflammation through their ability to inhibit pro-inflammatory enzymes such as COX-2 [76]. Polyphenols have also been suggested to inhibit platelet aggregation, which lowers the risk of excessive clotting, and to help prevent hyper-homocysteinemia, which has been linked to increased CVD risk [73]. In addition, the antioxidant effect of polyphenols is often explored; one RCT using hesperidin supplementation in 64 participants with type 2 diabetes found that it reduced oxidative DNA damage and lipid peroxidation, implying an effect on vascular function and lipid accumulation [77].

#### 5.2.2. Evidence for Optimal Intakes

The data from the current evidence on CVD suggests a possible range of intakes which could provide a beneficial effect to vascular health or at least seems to demonstrate no negative effects or consequences. Looking at observational studies, one prospective cohort study of more than 34,000 adults from the Netherlands, found that drinking up to one 150 mL glass per day of FJ was significantly associated with a 12–17% reduced risk of CVD and CHD, and a 20–24% reduced risk of stroke [25]. The previously mentioned SRMA by D’Elia et al., 2020 found that beneficial associations were seen at daily intakes of 100–200 mL, which is consistent with health guidance in several European countries [34]. Data from RCTs suggests that short-term intakes of up to 500 mL per day can provide benefits, but the longer-term effects of intakes this high are yet to be determined.

### 5.3. Cognitive Function

FJ compounds that have been associated with cardiovascular health would also be expected to impact on brain and cognitive health since CVD is a risk factor for certain types of dementia [78]. Metabolites of certain FJ compounds, such as polyphenols, can cross the blood–brain barrier enabling them to have a positive impact on cognitive function [79]. Some mechanisms for the effects of citrus polyphenols on brain health have been proposed based on animal studies and pre-clinical trials, for example promoting cerebral blood flow and increasing blood nitric oxide levels and oxygenation, as well as protecting brain cells from oxidative damage [78]. Effects on inflammation have also been proposed through the potential of FJ polyphenols to inhibit inflammatory signalling in glial cells, thus reducing the risk of neuroinflammatory injury [80].

No SRMAs on FJ and cognitive function have been published and RCTs are scarce—the systematic review by Pontifex et al., 2021 reviewed the findings of 10 human studies (4 RCT) on citrus flavanones observing that these were generally supportive of the animal and pre-clinical studies which reported cognitive performance improvements and lower disease risk [78]. Another systematic review focussed on fruits, vegetables and their juices, reporting that 17 out of 19 observational studies and three out of six intervention studies reported significant improvements in cognitive performance at higher intakes [81]. Greater benefits were seen for chronic consumption in healthy older adults.

Turning to specific RCTs, an acute crossover trial found that a berry smoothie maintained cognitive function in young adults during 6 h of fatiguing tests compared with an energy-matched control drink which resulted in cognitive decline [82]. Another acute RCT in young adults reported increased cerebral blood flow and improved cognitive performance in a test for speed, sustained attention and visual spatial skills following a single serving of flavonoid-rich orange juice relative to placebo [83]. In healthy older adults, an 8-week crossover RCT found that daily flavanone-rich orange juice significantly increased global cognitive function tests, while an acute RCT in middle-aged men concluded that a single serving of flavonoid-rich orange juice significantly improved executive function, alertness and psychomotor speed compared with placebo [84,85]. A 12-week crossover RCT in healthy, middle-aged women reported that a daily serving of purple grape juice significantly improved spatial memory and driving performance relative to placebo [86]. Finally, a 12-week RCT in older adults revealed that cognitive performance in a battery of tests improved after daily consumption of tart cherry juice compared with placebo [87]. Visual sustained attention and spatial working memory also significantly improved compared with baseline in the cherry juice group.

Further research is now required to test the cognitive impact of FJ polyphenols in longer-term human trials, especially in healthy older populations, and examine inter-relationships with the gut microbiota which are known to influence polyphenol bioavailability and certain disease models (e.g., lipopolysaccharide-induced neuroinflammation and dysfunction of the blood–brain barrier).

As human research is currently limited, it is not possible to speculate on optimal intakes for cognitive function.

## 6. Discussion

Even though FJ is classified as a source of free sugars by the WHO, and as such will form part of individual country targets for free sugars, it is nevertheless clear from the available evidence that FJs are not the same in terms of nutritional composition nor potential health effects as SSBs. As evidenced by SRMAs, SSBs are associated with weight gain, risk of obesity and risk of developing type 2 diabetes—but FJs are not. In addition, while FJs are associated with lower risk of CVD and improved vascular function in SRMAs and individual RCTs, these beneficial effects are not seen for SSBs.

A recent secondary analysis used data from the European Prospective Investigation into Cancer and Nutrition (EPIC-NL) cohort comprising >35,000 participants to examine the effects of SSBs, FJ and whole fruit on disease risk [88]. The findings from substitution analyses revealed that swapping 75–100% of total SSBs plus FJ for FJ alone lowered the hazard ratio to 0.74 for type 2 diabetes and to 0.85 for CHD, suggesting the benefit of reducing SSBs intake and replacing it with FJ. Further analyses found that substitution of whole fruit for FJ did not increase the risk of type 2 diabetes, CVD, CHD or stroke, suggesting a health impact equivalence of whole fruit and its juice. The previously mentioned SRMA of observational studies by Zurbau et al., 2020 supports this finding, with FJ consumption associated with a greater reduction in stroke mortality risk than either whole fruit or vegetables, and reductions in heart disease mortality being equivalent between the three food types [68].

The apparent differences between FJ and SSBs, often despite a similar total and free sugar content, are most likely due to the naturally occurring micronutrients and plant bioactives found in FJ. These include potassium, vitamin C, folate, carotenoids and the polyphenols: hesperidin, quercetin, carotenoids, anthocyanins and punicalagin. Such components, according to systematic reviews [74,78] and individual studies [17,41,72,89], are likely to explain the improved FMD and pulse pressure, lower BP, slower absorption of dietary sugars in the gut, and anti-inflammatory and antioxidant effects seen in RCTs when FJ is consumed regularly. The low GI of FJ (41–52) compared with that of SSBs (63–68) may be why FJ consumption seems to have no impact on glycaemic control in RCTs, or are associated with risk of type 2 diabetes despite a free sugar level of approximately 9–10 g/100 mL.

In terms of vascular effects, polyphenols are also likely to play a role. For example, orange juice consumption has the potential to increase nitric oxide (NO) plasma concentration, which could result in lower BP since changes in vascular function were positively correlated with plasma concentrations of flavanones in one RCT [89]. The BP-lowering effects of FJ could also involve an inhibitory effect on angiotensin-converting enzyme [90]. Potassium in FJ may also be a contributing factor since the relationship between potassium and BP is well researched, with a SRMA reporting that higher intakes of potassium are associated with decreased risk of stroke [74]. This effect is more pronounced in adults with hypertension or elevated sodium intake [74]. The rich vitamin C content of FJ could also have vascular effects through the regulation of prostacyclin production and NO activity [91].

## 7. Conclusions

The purpose of this review was to weigh up the evidence from studies investigating a potential health harm for FJ against evidence from studies which indicate a potential health benefit. Despite its classification as a source of free sugars, FJ appears to be associated with benefits rather than risks, especially in relation to vascular health and nutrient adequacy. FJ is an important, bioavailable source of polyphenols, which, in their own right, have been linked with health benefits. There is a suggestion that, for some health markers at least, a U-shaped curve exists, supporting moderate consumption of FJ (75–224 mL daily) consistent with the public health advice provided in the US dietary guidelines and those of several European countries where a serving of FJ counts towards daily fruit intakes. In contrast, there seems to be no justification for discouraging moderate consumption of FJ within a balanced diet or classifying FJ as a sugary beverage alongside SSBs.

## Figures and Tables

**Table 1:** Comparison of the nutritional composition of orange juice, whole orange and a typical sugar-sweetened beverage.

Per 100 Grams	Orange Juice	Whole Orange	Typical Sugar-Sweetened Beverage
Energy kcal	41	36	40.6
Total sugars g	9.1	8.2	10.8
Calcium mg	11	24	5.9
Iron mg	0.2	0.1	0
Magnesium mg	9.5	8	1
Phosphorus mg	15.3	16	29.7
Potassium mg	152	122	1
Zinc mg	0.1	Tr	0
Vitamin C mg	45	52	0
Thiamin mg	0.1	0.2	0
Riboflavin mg	0	0.03	0
Niacin mg	0.3	0.5	0
Folate mcg	21.5	33	0
Vitamin B6 mg	0.1	0.05	0
Vitamin A mcg	4.1	9	0
Vitamin E mcg	0.2	0.4	0
Total carotenoids mg	0.7	0.33	0
Hesperidin and narirutin mg	63	269	0
Pectin (fibre) mg	33.4	239	0
Fibre (AOAC) g	0.2–0.5	1.2	0

Sources of data [8,9,10,11,12].

## Data Availability

Not applicable.
